# The *Bordetella* Secreted Regulator BspR Is Translocated into the Nucleus of Host Cells via Its N-Terminal Moiety: Evaluation of Bacterial Effector Translocation by the *Escherichia coli* Type III Secretion System

**DOI:** 10.1371/journal.pone.0135140

**Published:** 2015-08-06

**Authors:** Akio Abe, Ryutaro Nishimura, Naomichi Tanaka, Jun Kurushima, Asaomi Kuwae

**Affiliations:** Laboratory of Bacterial Infection, Graduate School of Infection Control Sciences, Kitasato University, Tokyo 108-8641, Japan; Universidad Nacional de La Plata., ARGENTINA

## Abstract

*Bordetella bronchiseptica* is genetically related to *B*. *pertussis* and *B*. *parapertussis*, which cause respiratory tract infections in humans. These pathogens possess a large number of virulence factors, including the type III secretion system (T3SS), which is required for the delivery of effectors into the host cells. In a previous study, we identified a transcriptional regulator, BspR, that is involved in the regulation of the T3SS-related genes in response to iron-starved conditions. A unique feature of BspR is that this regulator is secreted into the extracellular milieu via the T3SS. To further characterize the role of BspR in extracellular localization, we constructed various truncated derivatives of BspR and investigated their translocation into the host cells using conventional translocation assays. In this study, the effector translocation was evaluated by the T3SS of enteropathogenic *E*. *coli* (EPEC), since the exogenous expression of BspR triggers severe repression of the *Bordetella* T3SS expression. The results of the translocation assays using the EPEC T3SS showed that the N-terminal 150 amino acid (aa) residues of BspR are sufficient for translocation into the host cells in a T3SS-dependent manner. In addition, exogenous expression of BspR in HeLa cells demonstrated that the N-terminal 100 aa residues are involved in the nuclear localization. In contrast, the N-terminal 54 aa residues are sufficient for the extracellular secretion into the bacterial culture supernatant via the EPEC T3SS. Thus, BspR is not only a transcriptional regulator in bacteria cytosol, but also functions as an effector that translocates into the nuclei of infected host cells.

## Introduction

The genus *Bordetella* is a Gram-negative aerobic coccobacilli that is currently subclassified into nine species [1]. Among them, *B*. *bronchiseptica*, *Bordetella pertussis*, and *B*. *parapertussis* share a large number of virulence factors, including toxins, adhesins, and components of the type III secretion system (T3SS) [2]. *B*. *parapertussis* has been classified into two distinct lineages, *B*. *parapertussis*HU and *B*. *parapertussis*OV, which cause respiratory tract infections in humans and sheep, respectively [3]. *B*. *pertussis* and *B*. *parapertussis*HU are strictly human-adapted pathogens and are the etiological agents of whooping cough (pertussis) in humans. In contrast, *B*. *bronchiseptica* causes chronic infections in a broad range of animals and has also been isolated from human immunodeficiency virus-infected patients [4].

To exert full virulence in the hosts, *Bordetella* coordinately regulates a number of virulence genes by a two-component signal transduction system, BvgA and BvgS (BvgAS) [5]. BvgS is a transmembrane sensor kinase that is autophosphorylated in response to environmental signals and then eventually transfers its phosphate group to the DNA-binding responsive activator BvgA [6]. The resulting activated BvgA is able to bind to promoter regions, leading to the transcriptional activation of a wide variety of virulence genes (Bvg+ phase) [7]. On the other hand, the expression of Bvg-induced genes was reduced when concentrations of MgSO_4_ in the culture medium were increased (Bvg^−^ phase). Thus, *Bordetella* virulence genes are coordinately regulated by the BvgAS system in response to various environmental conditions.

Components of the T3SS, regulators, and secreted proteins are encoded in the T3SS-related gene cluster, the *bsc* locus, which consists of 29 genes [8]. The *btr* locus is located adjacent to the *bsc* locus and is involved in the regulation of the T3SS-related genes at the transcriptional or post-transcriptional level [8]. In *Bordetella*, BopB, BteA (also referred to as BopC), BopD, BopN, Bsp22, and BspR have been identified as type III secreted proteins [9–15]. Bsp22 polymerizes and assembles into filaments at the tip of the T3SS needle structure and interacts with BopD [13]. BopB and BopD are delivered into the host membrane via the T3SS and form a translocation pore complex that functions as a conduit for effectors [11,12]. Two type III effectors, BteA and BopN, are involved in the induction of necrosis in various types of mammalian cells [10,15] and production of IL-10, an anti-inflammatory cytokine [14], respectively.

As described in detail previously [9], BspR negatively regulates the expression of genes encoding the type III secreted proteins at the transcriptional level. In contrast, the expression of genes encoding filamentous hemagglutinin (FhaB), protection (Prn), and CyaA is positively regulated by BspR [9]. Interestingly, the expression of T3SS-related genes in *Bordetella* is activated under iron-starved conditions [16,17] and type III secreted proteins were aberrantly induced by the BspR mutant [9], suggesting that the iron-responsive modulation is involved in the BspR-mediated T3SS regulation. Furthermore, proteomic analysis has shown that the production of BvgA in the *bspR* mutant was significantly higher than that in the *B*. *bronchiseptica* wild type [9]. Thus, BspR acts as a molecular switch for a large number of virulence genes via alteration of BvgA levels in the bacterial cytosol.

Recently, we demonstrated that BspR is secreted into bacterial culture supernatants via the T3SS [9]. While BspR functions as a regulator in bacterial cytosol, the extracellular properties of BspR remain to be elucidated. To further characterize the function of BspR, we constructed various truncated derivatives of BspR and investigated their translocation into the host cells. Herein, we report that BspR is translocated into the host cells via the T3SS and has the ability to localize into the nucleus.

## Materials and Methods

### Bacterial strains, and growth media

The strains used in this study are listed in Table 1. The inoculum of *Bordetella* strain was prepared from fresh colonies grown on Bordet and Gengou (BG) agar, as described previously [9,18,19], and were cultured in Stainer and Scholte (SS) medium, with a starting *A*_600_ of 0.2 under vigorous shaking at 37°C. The liquid cultivation period was 18 h for the protein preparation experiments and infection assays. EPEC strains were inoculated in lysogeny broth (LB) and incubated at 37°C for 16 h without shaking. The overnight culture was diluted (1:25) in Dulbecco’s modified Eagle’s medium (DMEM) (Sigma), and the EPEC cultures were grown at 37°C under an atmosphere of 5% CO_2_ for 6 h without shaking.

**Table 1:** Strains used in this study.

Strain	Genotype/description	Reference of source
S798	Wild-type *B*. *bronchiseptica*	[12]
S798 Δ*bspR*	S798 Δ*bspR*	[9]
S798 Δ*bteA* Δ*cyaA*	S798 Δ*bteA*, Δ*cyaA*	This study
S798 ΔT3SS	S798 Δ*bscN* (mutated for ATPase driving the T3SS)	[12]
E2348/69	Wild-type EPEC O126	[33]
E2348/69 Δ*espB*	EPEC Δ*espB*	[34]
E2348/69 ΔT3SS	EPEC Δ*escF* (mutated for the T3SS needle structure)	[20]
DH10B	F^−^ *mcrA* Δ(*mrr*-*hsdRMS*-*mcrBC*) Φ80*lacZ*ΔM15 Δ*lacX*74 *recA1 endA1 araD139* Δ(*ara*, *leu*)7697 *galU galK rpsL deoR nupG* λ^−^	Invitrogen
Sm10λ*pir*	Permissive strain for replication of pCVD442	[35]

### Cell culture and transfection

COS-7 (ATCC CRL-1651) and HeLa (ATCC CCL-2) cells were maintained in DMEM and Eagle’s minimum essential medium (EMEM) (Sigma), respectively, each containing 10% fetal calf serum, at 37°C under an atmosphere of 5% CO_2_. COS-7 and HeLa cells were seeded in a 6-well plate at 5 × 10^5^ cells/well and incubated for 20 h, and were transfected with 2.5 μg/ml pCAG-BspR-FL, pCAG-BspR-NT, and pCAG-BspR-CT using Lipofectamine LTX (Invitrogen) according to the manufacturer’s instructions. After transfection, the cells were incubated at 37°C for 24 h and fixed in 4% paraformaldehyde.

### Construction of the *cyaA* mutant

pDONR221 (Invitrogen) and pABB-CRS2 [20] were used as cloning and positive suicide vectors, respectively. The construction of a deletion mutant using pABB-CRS2 has been described previously [10]. A 7.1-kbp DNA fragment containing the *cyaA* gene and its flanking region was amplified by PCR with the primers B1-*cyaA* and B2-*cyaA* using *B*. *bronchiseptica* S798 genomic DNA as a template. The resulting PCR product was cloned into pDONR221 using the adaptor PCR method (Gateway cloning system, Invitrogen) to obtain pDONR-*cyaA*. For the deletion of *cyaA* in pDONR-*cyaA*, inverse PCR was then carried out with the primers R1-*cyaA* and R2-*cyaA* using circular pDONR-*cyaA* as a template. The resulting PCR products were digested with HindIII and self-ligated to obtain pDONR-Δ*cyaA*, which contained a HindIII site in addition to a 4959-bp deletion including the start codon of *cyaA*. The *cyaA* fragment with internal deletion was transferred to pABB-CRS2 to obtain pABB-CRS2-Δ*cyaA* using the Gateway cloning system. pABB-CRS2-Δ*cyaA* was then introduced into *E*. *coli* SM10λ*pir* and was transconjugated into *B*. *bronchiseptica* S798 Δ*bteA*, which is unable to induce necrosis to mammalian cells as described previously [10]. The resulting mutant strain was designated S798 Δ*bteA* Δ*cyaA*.

### Preparation of proteins from culture supernatants and whole bacterial cells

Proteins secreted into the bacterial culture supernatants, and the whole bacterial cell lysates were isolated by trichloroacetic acid precipitation, as described previously [10]. Culture supernatants were filtered, and bacterial pellets were resuspended in distilled water. Trichloroacetic acid was added to the respective samples at a final concentration of 10%. After incubation of the samples on ice for 15 min, they were centrifuged for 5 min. The resulting precipitated proteins were neutralized with 2 M Tris-base and dissolved in SDS-polyacrylamide gel electrophoresis sample buffer.

### Primers and plasmids

The primers and the plasmids used in this study are listed in Tables 2 and 3, respectively.

**Table 2:** Primers used in this study.

Primer	Sequence
*att*B1	5'-GGGGACAAGTTTGTTTGTACAAAAAAGCAGGCT-3'
*att*B1r	5’-GGGGACTGCTTTTTTGTACAAACTTGN-3’
*att*B2	5'-GGGGACCACTTTGACAAGAAAGCTGGGT-3'
*att*B4	5’-GGGGACAACTTTGTATAGAAAAGTTGNN
5-*cyaA*-gs	5'-GGTGGCGGTTCTCAGCAATCGCATCAGGCTGG-3'
B1-*cyaA*	5’-AAAAAGCAGGCTGCGGCCGCGTCCAGCGCGCG-3’
B2-*cyaA*	5’-AGAAAGCTGGGTTGACGGCGATGAACACGACG-3’
R1-*cyaA*	5’-CGCAAGCTTGGGGATGCCAGACTCCCGGTC-3’
R2-*cyaA*	5’-GCCAAGCTTGCAATGGCGCAGTACCCGGAC-3’
B2-*cyaA*	5'-AGAAAGCTGGGTCTAGTCATAGCCGGAATCCTGGC-3'
B1-*bspR*-N1	5'-AAAAAGCAGGCTACCCAAGGATTCGAGACAAC-3'
B4F-*dnt*P	5'-ATAGAAAAGTTGTTGGCCGGGCACACCCGCAAC-3'
B1R-*dnt*P	5'-TGTACAAACTTGGTTTGTTTACCCCTGACCGG-3'
*bspR*-5aa	5'-CTGAGAACCGCCACCGATCTGGAAGTTCATGCGGTG-3'
*bspR*-10aa	5'-CTGAGAACCGCCACCAGGTAAAGCGGGTGGGATCTG-3'
*bspR*-54aa	5'-CTGAGAACCGCCACCGGCGACCCAGGATACGTTGC-3'
*bspR*-100aa	5'-CTGAGAACCGCCACCTCGCGTGACGACACGCGCCG-3'
*bspR*-150aa	5'-CTGAGAACCGCCACCCAACGTTCCGGATATCTGATC-3'
5-*bspR*-NdeI	5'-GGAATTCCATATGACTCTCCGCGTTGACGGCGC-3'
3-*bspR*-KpnI	5'-GGGGTACCCAGGTGGTGCGCAAGGACCT-3'
B3R-*rrnB*	5'-ATAATAAAGTTGAAACAAAAAGAGTTTGTAGAAACG-3'
3-IF-*bspR*	5’-TAGCAAGCTTCTCGAGCATCAGGTGGTGCGCAAGGACCTCG-3’
5-IF-*bspR*	5’-GCGAGTCTTCGAATTCGCCACCATGGACTTCCAGATCCCACCCGC-3’
*bspR*-100-FL	5’-AGCAAGCTTCTCGAGTCGCGTGACGACACGCGCCG-3’
*bspR*-100-FU	5’-CTCGAGAAGCTTGCTAGCAG-3’
*bspR*-191-FL	5’-CATGGTGGCGAATTCGAAGAC-3’
*bspR*-191-FU	5’-GAATTCGCCACCATGGCCGTCGACATGGCGGAAAC-3’

**Table 3:** Plasmids used in this study.

Plasmid	Description	Reference or sourse
pDONR201	Gateway cloning vector	Invitrogen
pDONR221	Gateway cloning vector	Invitrogen
pDONR P4-P1R	Gateway cloning vector	Invitrogen
pABB-CRS2	Suicide vector	[20]
pDONR-*cyaA*	*cyaA* cloned into pDONR221	This study
pDONR-Δ*cyaA*	*cyaA* deletion cloned into pDONR221	This study
pABB-CRS2-ΔcyaA	*cyaA* deletion cloned into pABB-CRS2	This study
pMS109	Plasmid containing *cyaA*	[36]
pDONR-BspR-FL	*bspR-cyaA* cloned into pDONR201	This study
pDONR-*fha*P	*fha* promoter region cloned into pDONR201	[10]
pDONR-*dnt*P	*dnt* promoter region cloned into pDONR201	This study
pDONR-*cya*P	*cyaA* promoter region cloned into pDONR201	This study
pDONR-*rrnB*	*rrnB* terminator region cloned into pDONR201	[10]
pRK-R4-R3-F	Expression vector for *Bordetella*	[10]
pBspR-FL	*bspR-cyaA* cloned into pRK-R4-R3-F	This study
pDONR-BspR-150	*bspR* truncated derivative for pDONR-BspR-FL	This study
pDONR-BspR-100	*bspR* truncated derivative for pDONR-BspR-FL	This study
pDONR-BspR-54	*bspR* truncated derivative for pDONR-BspR-FL	This study
pDONR-BspR-10	*bspR* truncated derivative for pDONR-BspR-FL	This study
pDONR-BspR-5	*bspR* truncated derivative for pDONR-BspR-FL	This study
pBspR-150	pBspR-FL derivative producing BspR (aa 1–150)—CyaA	This study
pBspR-100	pBspR-FL derivative producing BspR (aa 1–100)—CyaA	This study
pBspR-54	pBspR-FL derivative producing BspR (aa 1–54)—CyaA	This study
pBspR-5	pBspR-FL derivative producing BspR (aa 1–5)—CyaA	This study
pABB-Trc99cm	*E*. *coli* expression vector	[20]
pABB-BspR-FL	*bspR*-*cyaA* cloned into pABB-Trc99cm	This study
pABB-BspR-150	pABB-BspR-FL derivative producing BspR (aa 1–150)—CyaA	This study
pABB-BspR-100	pABB-BspR-FL derivative producing BspR (aa 1–100)—CyaA	This study
pABB-BspR-54	pABB-BspR-FL derivative producing BspR (aa 1–54)—CyaA	This study
pABB-BspR-10	pABB-BspR-FL derivative producing BspR (aa 1–10)—CyaA	This study
pABB-BspR-5	pABB-BspR-FL derivative producing BspR (aa 1–5)—CyaA	This study
pCX340	TEM 1 fusion vector	[21]
pCX340-BspR	*bspR* cloned into pCX340	This study
pCX340-Map	EPEC *map* cloned into pCX340	[22]
pCX340-CesT	EPEC *cesT* cloned into pCX340	[22]
pCAG-MCS2-FOS	Mammalian expression vector producing C-terminal FOS-tagged fusion protein	[37]
pC-BspR-FL	Full length *bspR* cloned into pCAG-MCS2-FOS	This study
pC-BspR-NT	pC-BspR-FL derivative for BspR (aa 1–100)	This study
pC-BspR-CT	pC-BspR-FL derivative for BspR (aa 101–191)	This study

### Construction of a plasmid for the production of BspR-CyaA fusion protein in *Bordetella*

A 0.6-kbp fragment encoding *bspR* was amplified by PCR with B1-*bspR*-N1 and 3-IF-*bspR* primers using S798 genomic DNA as a template. A DNA fragment encoding the catalytic domain (N-terminal 400 amino acid residues) of *B*. *pertussis* CyaA was amplified with 5-*cyaA*-gs and B2-*cyaA* primers using pMS109 as a template. Both *bspR* and *cyaA* fragments were ligated together using In-Fusion enzyme (Clontech) and then *att*B1 and *att*B2 recognition sites were added to the 5' and 3' flanking sites of the *bspR*-*cyaA* fused gene, respectively, using the adaptor PCR method in the Gateway cloning system (Invitrogen). The resulting *bspR*-*cyaA* was cloned into pDONR201 to obtain pDONR-BspR-FL by BP Clonase reaction in the Gateway cloning system (Invitrogen). The promoter region of *dnt* was amplified by PCR with B4F-*dntP* and B1R-*dntP* primers using S798 genomic DNA as a template, and then *att*B4 and *att*B1r sites were added to the 5' and 3' flanking sites of the *dnt* promoter region, respectively, using the adaptor PCR method. The resulting PCR product was inserted into pDONR P4-P1R by BP Clonase reaction to obtain pDONR-*dnt*P. To allow expression of the *bspR-cyaA* fusion gene under the control of the *dnt* promoter and the *rrnB* terminator, pDONR-*dnt*P, pDONR-BspR-FL, pDONR-*rrnB*, and *Bordetella* vector pRK415 R4-R3-F were mixed and treated with LR Clonase Plus (Invitrogen) to clone the *dnt* promoter, *bspR-cyaA* gene, and *rrnB* terminator into pRK415 R4-R3-F using the MultiSite Gateway system (Invitrogen), and the resulting plasmid was designated pBspR-FL.

### Construction of plasmids for BspR-domain mapping in *Bordetella*

To obtain plasmids encoding truncated versions of BspR fused with CyaA, inverse PCR was performed with the primers 5-*cyaA*-gs and *bspR*-150aa using circular pDONR-BspR-FL as a template to obtain pDONR-BspR-150 that produces the N-terminal 150 aa residues of BspR fused with CyaA. Using the same experimental procedure, pDONR-BspR-100 (N-terminal 100 aa residues of BspR fused with CyaA), pDONR-BspR-54 (N-terminal 54 aa residues of BspR fused with CyaA), pDONR-BspR-10 (N-terminal 10 aa residues of BspR fused with CyaA) and p DONR-BspR-5 (N-terminal 5 aa residues of BspR fused with CyaA), were also constructed using the primer sets, 5-*cyaA*-gs and *bspR*-100aa, 5-*cyaA*-gs and *bspR*-54aa, 5-*cyaA*-gs and *bspR*-10aa, and 5-*cyaA*-gs and *bspR*-5aa, respectively. Using the MultiSite Gateway system, pDONR-BspR-150, pDONR-BspR-100, pDONR-BspR-54, and pDONR-BspR-5 were mixed with pDONR-*dnt*P, pDONR-*rrnB*, and pRK415 R4-R3-F in the presence of LR Clonase to obtain pBspR-150, pBspR-100, pBspR-54, and pBspR-5, respectively.

### Construction of plasmids for BspR-domain mapping in *E*. *coli*

To allow expression of the full length *bspR* and its truncated derivatives fused with the cyaA, pDONR-BspR-FL, pDONR-BspR-150, pDONR-BspR-100, pDONR-BspR-54, pDONR-BspR-10, and pDONR-BspR-5 were mixed with the *E*. *coli* expression vector pABB-Trc99cm in the presence of LR Clonase to obtain pABB-BspR-FL, pABB-BspR-150, pABB-BspR-100, pABB-BspR-54, and pABB-BspR-5, respectively. For the expression of BspR fused with TEM-1 β-lactamase, *bspR* was cloned into the plasmid pCX340 [21] as follows. An 896 bp fragment encoding *bspR* was amplified by PCR with the primers 5-*bspR*-NdeI and 3-*bspR*-KpnI using S798 genomic DNA as a template. The resulting fragment was digested with NdeI and KpnI and then cloned into similarly digested pCX340. The resulting plasmid was designated pCX-BspR. The plasmids pCX-Map and pCX-CesT have been described previously [22].

### Construction of plasmids for the BspR expression in mammalian cells

To allow expression of BspR-FLAG in mammalian cells, a DNA fragment encoding *bspR* was amplified by PCR with 3-IF-*bspR* and 5-IF-*bspR* primers using S798 genomic DNA as a template, and the resulting fragment was digested with EcoRI and XhoI and then cloned into similarly digested pCAG-MCS2-FOS to obtain pC-BspR-FL. To obtain plasmids encoding the N-terminal half (1–100 aa) of BspR, inverse PCR was performed with the primers *bspR*-100-FU and *bspR*-100-FL using circular pC-BspR-FL as a template to obtain pC-BspR-NT. Using the same experimental procedure, pC-BspR-CT (C-terminal half; 101–191 aa) was also constructed by inverse PCR using the primers *bspR*-191-FU and *bspR*-191-FL.

### Antibodies and immunoblot analysis

The anti-BopB antibodies used in this study have been described previously [12]. The anti-CyaA antibodies were purchased from Santa Cruz Biotechnology. The anti-TEM-1 β-lactamase antibodies were purchased from QED bioscience Inc. The anti-FLAG antibodies were purchased from Sigma. The secondary antibodies conjugated to HRP were Protein A (GE Healthcare) and anti-mouse IgG (Sigma). For the immunoblot analysis, protein samples were resolved by SDS-PAGE and transferred to polyvinylidene difluoride membranes (Immobilon filter; Millipore). The proteins on the membrane were detected by immunoblotting using a Luminata Forte Western HRP substrate (Millipore) and the digital imaging system ImageQuant LAS 4000 (GE Healthcare). Quantification was achieved with the ImageQuant TL software (GE Healthcare Life Sciences).

### Fluorescence staining

COS-7 cells were fixed for 15 min with 4% paraformaldehyde in PBS and subjected to immunofluorescence staining with anti-FLAG antibodies. As a secondary antibody, Alexa Fluor 488 goat anti-mouse IgG (Invitrogen) was used. Nuclei were stained with DAPI (Invitrogen). Numbers of FLAG-positive nuclei were scored by examining 100 cells per coverslip under a fluorescence microscope.

### TEM-1-based translocation assay

The TEM-1-based translocation assay is applicable for the evaluation of effector translocation using living host cells [21]. The nonfluorescent CCF2/AM substrate is converted to fluorescent CCF2 by the cellular esterases, and the resulting CCF2 emits a green fluorescence signal at 520 nm. The effector-mediated delivery of the TEM-1-fusion protein into the host cells induces the catalytic cleavage of the lactam ring of CCF2, leading to a detectable change of the CCF2 fluorescence from green to blue emission. A translocation assay was performed as described previously [22]. HeLa cells seeded at 4 x 10^5^ cells/well on coverslips in a 6-well plate with DMEM (Sigma) and 10% fetal calf serum were infected with precultured EPEC in DMEM at an MOI of 100. The cells were then centrifuged for 5 min and incubated at 37°C in an atmosphere of 5% CO_2_. After 30 min, isopropyl-β-D-thiogalactopyranoside (IPTG) was added to the cultures at a final concentration of 1 mM, and the infection was allowed to proceed for an additional 1 h. The infected cells were then washed with PBS and stained with a CCF2/AM loading kit (Invitrogen). The cells were then observed with a fluorescence microscope equipped with a 4’,6’-diamidino-2-phenylindole (DAPI) filter set (365-nm excitation and 397-nm long-pass emission filters).

### CyaA-based translocation assay

The CyaA-based translocation assay relies on the effector-mediated delivery of the CyaA-fusion protein into the host cytosol, where the CyaA moiety is activated by association with cytosolic calmodulin, leading to increases in cAMP [23]. Intracellular cAMP can be measured using the cAMP enzyme immunoassay. Translocation of the CyaA fusion protein by T3SS into infected host cells was measured using a cyclic AMP (cAMP) enzyme immunoassay (Amersham cAMP Biotrak Enzymeimmunoassay System; GE Healthcare Life Sciences) to quantify intracellular cAMP levels. EPEC strains carrying the plasmids encoding the BspR-CyaA fusion proteins were inoculated in LB medium and incubated at 37°C for 16 h without shaking. HeLa cells (4 × 10^4^ cell/well) were infected with EPEC strains at an MOI of 100 for 3 h in the presence of IPTG at a final concentration of 1 mM. After infection, cells were lysed and treated according to the manufacturer's instructions.

### Detection of BspR in infected COS-7 Cells

A mammalian vector carrying BspR-FLAG fusion protein was introduced into COS-7 cells. To detect BspR-FLAG expression in COS-7 cells, the transfected COS-7 cells were washed three times with ice-cold PBS, and the cells were then treated with lysis buffer [24]. The cell lysates were sonicated for 30 s and separated by centrifugation at 15,000 *g* for 15 min. The supernatants were then separated by SDS-PAGE and the specific BspR-FLAG signal was detected by immunoblot analysis using anti-FLAG antibodies.

## Results

### The N-terminus of BspR is involved in the negative regulation of T3SS

**Fig 1:**
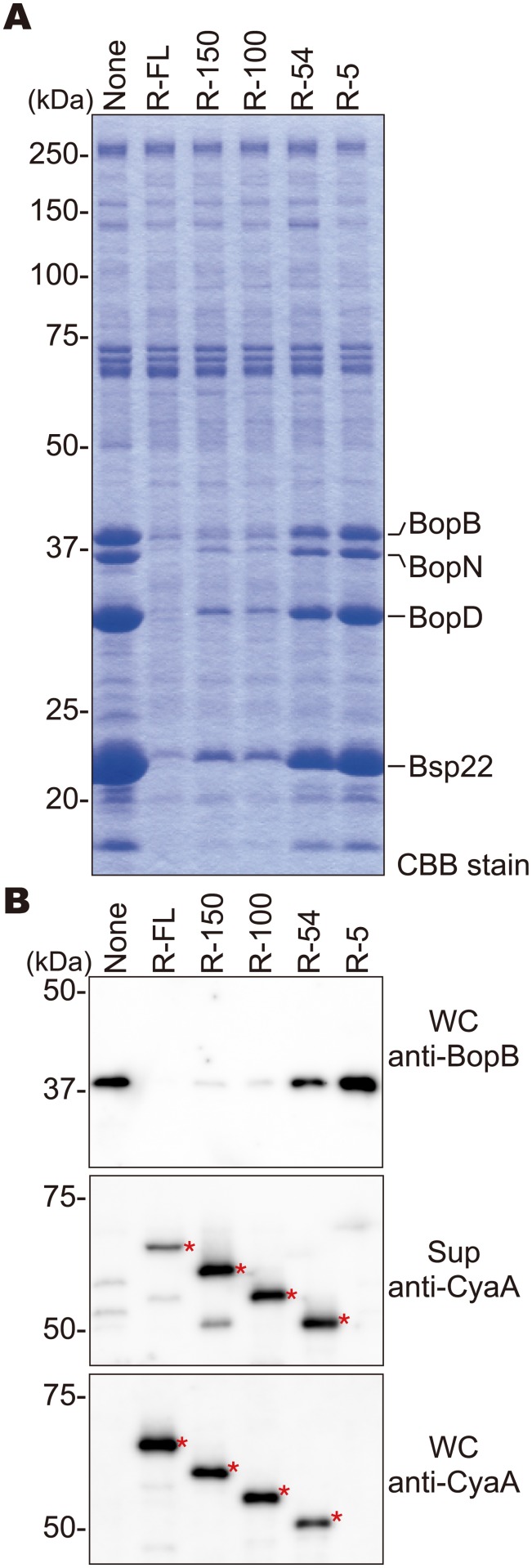
The N-terminal region of BspR is associated with secretion signal and negative regulation of T3SS. *B*. *bronchiseptica* S798 strains carrying expression vectors for the production of BspR-CyaA and its derivatives were grown in SS medium under vigorous shaking at 37°C for 18 h. **(A)** The secreted proteins prepared from the culture supernatants were resolved by SDS-PAGE and stained with CBB. BopB, BopN, BopD, and Bsp22 were shown to be secreted into culture supernatants via the T3SS. **(B)** The secreted protein fractions in culture supernatants (Sup) and whole-cell lysates (WC) were isolated from the bacterial cultures and validated by immunoblot analysis using anti-BopB and anti-CyaA antibodies. Asterisks indicate the positions of the signals specific for each BspR-CyaA fusion protein. Secreted proteins and whole-cell lysates were prepared from *B*. *bronchiseptica* S798 harboring no-plasmid (None), pBspR-FL (R-FL; full length BspR), pBspR-150 (R-150; aa 1–150), pBspR-100 (R-100; aa 1–100), pBspR-54 (R54; aa 1–54), or pBspR-5 (R-5; aa 1–5).

In our previous study, we demonstrated that BspR is secreted into bacterial culture supernatants via the T3SS and functions as a transcriptional regulator in *B*. *bronchiseptica* [9]. To determine whether BspR is translocated into the host cells via the T3SS, plasmids carrying the full length BspR or its truncated derivatives fused with CyaA were constructed and introduced into the *B*. *bronchiseptica* S798 Δ*bteA* Δ*cyaA* strain, which is deficient in the induction of host cell death by the effector BteA [10] and lacks endogenous CyaA activity (S1 Fig). We would expect that the translocation of BspR-CyaA into the host cells could be detected by using the resulting S798 Δ*bteA* Δ*cyaA* strains containing recombinant plasmids and a quantitative cAMP assay. To confirm the expression of BspR-CyaA in the S798 Δ*bteA* Δ*cyaA* strain, the secreted proteins were prepared from the culture supernatants and analyzed by sodium dodecyl sulfate polyacrylamide gel electrophoresis (SDS- PAGE) and Coomassie brilliant blue (CBB) staining (Fig 1A). We detected the type III secreted proteins, BopB, BopD, BopN, and Bsp22, in culture supernatants from the S798 Δ*bteA* Δ*cyaA* strain with or without the plasmids. However, secretion of the type III secreted proteins was greatly reduced in the S798 Δ*bteA* Δ*cyaA* strain by the introduction of pBspR-FL, pBspR-150, and pBspR-100, but not pBspR-54 or pBspR-5. To further characterize the effect of the BspR-CyaA fusion protein on the T3SS, whole bacterial lysates and culture supernatants were prepared and assessed by immunoblotting (Fig 1B). Again, the production of BopB was greatly reduced in the S798 Δ*bteA* Δ*cyaA* strain by the introduction of pBspR-FL, pBspR-150, and pBspR-100. In addition, the production of BopB was partially reduced by introduction of pBspR-54 into the above strain. We also confirmed that the signals specific for each of the BspR-CyaA fusion proteins were detected in the strains harboring pBspR-FL, pBspR-150, pBspR-100, and pBspR-54, but not pBspR-5 (Fig 1B). Collectively, these results indicate that the BspR-CyaA fusion protein is secreted into bacterial culture supernatants and the extracellular secretion signal is included in the N-terminal 54 aa residues of BspR. Moreover, the N-terminal 100 aa residues of BspR was found to be involved in the negative regulation of T3SS (Fig 1). The expression of the T3SS-related genes was not affected by the endogenous expression of BspR when the S798 Δ*bteA* Δ*cyaA* strain was cultured in SS media containing 36 μM of iron (Fig 1, lane: None). In contrast, the exogenous expression of BspR resulted in stringent repression of the T3SS function by the gene dosage effect even under the same culture conditions (Fig 1, lane: R-FL, R150, or R-100). In particular, secretion of the translocators, BopB, BopD, and Bsp22, was greatly reduced by the exogenous expression of BspR (Fig 1A), resulting in blockade of the effector translocation. For this reason, we could not evaluate the translocation of BspR into the host cells in the *B*. *bronchiseptica* S798 Δ*bteA* Δ*cyaA* strain (S1 Fig).

### BspR is translocated into the host cells via the EPEC T3SS

**Fig 2:**
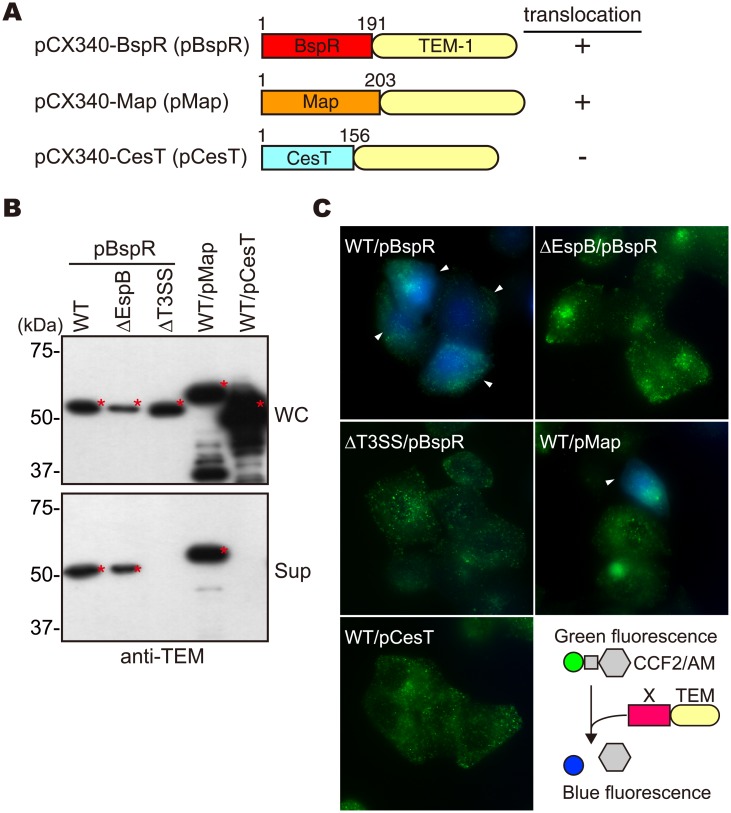
Translocation of BspR into host cells via the T3SS in enteropathogenic *E*. *coli*. **(A)** Construction of expression vectors for TEM-1 fusion proteins and the results of the translocation assays. **(B)** Whole-cell lysates (WC, upper panel) and secreted proteins in the supernatants (Sup, lower panel) isolated from each EPEC strain harboring the indicated plasmid were validated by immunoblotting using anti-β-lactamase (TEM-1) antibodies. Asterisks indicate the positions of the signals specific for each TEM-1 fusion protein. **(C)** HeLa cells were infected with the indicated strains and then stained with CCF2-AM solution. Stained cells were analyzed under a fluorescence microscope. Arrowheads show the fluorescence signal shift to blue from green, which indicates the cleavage of cytoplasmic CCF2 by the translocation of TEM-1 β-lactamase into the host cells.

In a previous study, we demonstrated that exogenous expression of an effector Map [25] of enteropathogenic *E*. *coli* (EPEC) was secreted into the culture supernatant and translocated into the host cells via the *B*. *bronchiseptica* T3SS [10]. Therefore, we would expect that the *Bordetella* effectors could be secreted and translocated into the host cells via the EPEC T3SS. For this reason, we constructed *E*. *coli* expression vectors for production of the full length BspR or its C-terminal deletions fused with a TEM-1 (Fig 2A). The BspR-TEM-1 and Map-TEM-1 fusion proteins were secreted into the culture supernatant in the wild-type EPEC, but not in the T3SS-deficient strain (Fig 2B). As expected, EPEC CesT [26], which is a chaperone for effector Tir and is localized in the bacterial cytosol, was not secreted into the culture supernatant, even in the wild-type EPEC. Next, HeLa cells were infected with EPEC carrying TEM-1 fusion proteins and the translocation of these TEM-1 fusion proteins was evaluated using a CCF2/AM substrate as described in the Materials and Methods (Fig 2C). The translocation of the BspR-TEM-1 fusion protein into the HeLa cells was detected for the EPEC wild-type strain, but not the T3SS-deficient mutant (Fig 2C). In contrast, we could not evaluate translocation of the BspR-TEM-1 fusion protein into the HeLa cells using *B*. *bronchiseptica* due to the unfavorable repression of the T3SS caused by the over-expression of BspR-TEM-1 (S2 Fig). Moreover, BspR-TEM-1 was secreted into the supernatant but no longer translocated into host cells in a strain lacking the translocator EspB (Fig 2B). Collectively, these data strongly suggest that BspR is translocated into the host cells via the T3SS.

### N-terminal BspR is required for translocation into the host cells

**Fig 3:**
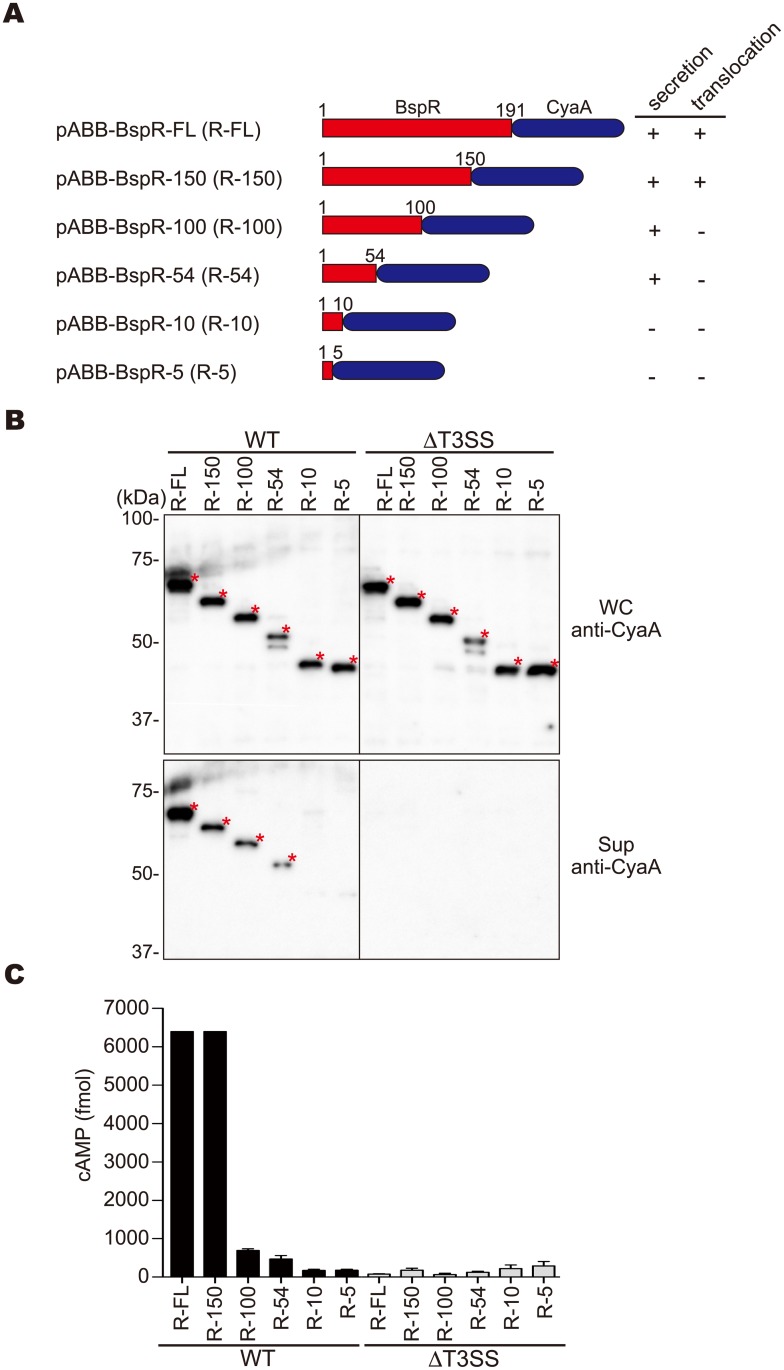
The N-terminus of BspR is involved in its translocation into host cells via T3SS. **(A)** Construction of the Cya fusion proteins used in the secretion and translocation assays. **(B)** Whole-cell lysates (WC, upper panel) and the fractions containing secreted proteins in the supernatants (Sup, lower panel) isolated from each EPEC strain harboring the indicated plasmids were analyzed by immunoblotting using anti-CyaA antibodies. Asterisks indicate the positions of the signals specific for each BspR-CyaA fusion protein. **(C)** HeLa cells were infected with the indicated strains and translocation of the CyaA fusion protein via T3SS into the infected cells was measured using a cAMP enzyme immunoassay. WT and ΔT3SS indicate the wild-type EPEC and the T3SS deficient strains, respectively. The values represent the means ± SD from three independent experiments.

The above data clearly demonstrate that the EPEC T3SS has the ability to deliver the *Bordetella* protein BspR into living host cells. To further analyze the responsible domain for the BspR translocation into the host cells, we constructed *E*. *coli* expression vectors for the production of the full length BspR or its C-terminal deletions fused with a CyaA (Fig 3A). The resulting constructs were introduced into the wild-type or the ΔT3SS EPEC strain, and whole bacterial lysates and bacterial culture supernatants were prepared and analyzed by immunoblotting using anti-CyaA antibodies (Fig 3B). All signals of the CyaA-fused proteins were detected in whole cell lysates at their deduced molecular masses (Fig 3B asterisks), and the band intensities were similar between the wild-type and ΔT3SS EPEC strains. In contrast, signals of BspR (R-FL) and the BspR truncated versions (R-150, R-100, and R-54) fused with CyaA were detected in the supernatant fractions prepared from the wild-type EPEC, but not from the ΔT3SS EPEC. These results strongly suggest that the BspR-CyaA fusion proteins were secreted into the bacterial culture supernatants, and the N-terminal 54 aa residues of BspR were sufficient for its secretion via the EPEC T3SS. We also confirmed that the EPEC T3SS was not affected by the exogenous expression of BspR (S3 Fig). Next, HeLa cells were infected with EPEC strains expressing BspR-CyaA fusion proteins and translocation of BspR-CyaA into the HeLa cells was evaluated using a quantitative cAMP assay. Elevated cAMP levels were detected in the HeLa cell lysates infected with the wild-type EPEC harboring pABB-BspR-FL or pABB-BspR-150 (Fig 3C). In contrast, detectable levels of cAMP were not observed in the ΔT3SS EPEC strains, even with the full length BspR fused with CyaA. Collectively, these results clearly indicate that BspR is translocated into the host cells in a T3SS-dependent manner and that the translocation signal is included in the N-terminal 150 aa residues of BspR.

### BspR is localized in the nuclei of the host cells

**Fig 4:**
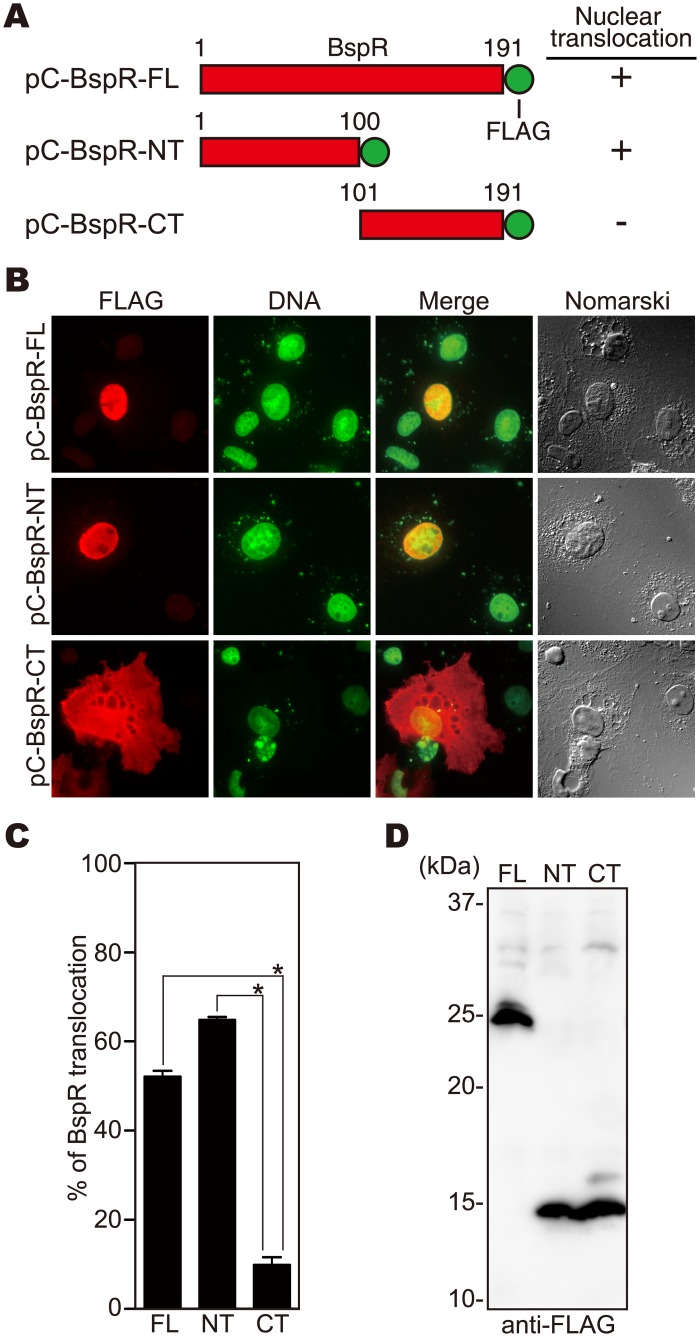
BspR is translocated into the nuclei of host cells. **(A)** Construction of the BspR tagged with FLAG used for the nuclear translocation experiments. **(B)** The mammalian expression vector for BspR production was introduced into COS-7 cells. 24 h after transfection, COS-7 cells were fixed and stained with anti-FLAG antibodies to detect BspR (red) and DAPI for nuclei (green). **(C)** The numbers of FLAG-positive nuclei were scored by examining 100 cells per coverslip under a fluorescence microscope. COS-7 cells transfected with pC-BspR-FL, pC-BspR-NT, and pC-BspR-CT were randomly picked from the immunofluorescence micrographs **(B)**, and the percentages of cells showing nuclear translocation of BspR were determined. The percentages were based on a count of 100 cells, and the values represent the means ± SD from three independent experiments. *, *p* < 0.05. **(D)** pC-BspR-FL (FL), pC-BspR-NT (NT), and pC-BspR-CT (CT) were introduced into COS-7 cells. 24 h after infection, the BspR-FLAG fusion protein was detected by immunoblot analysis of COS-7 cell lysate samples.

To further characterize the localization of BspR in the infected host cells, we constructed mammalian expression vectors for the production of BspR fused with the FLAG-tag as follows; full length BspR (pC-BspR-FL; aa 1–191); N-terminal half (pC-BspR-NT; aa 1–100); and C-terminal half (pC-BspR-CT; aa 101–191) (Fig 4A). The resulting constructs were introduced into COS-7 cells. Interestingly, the full length and the N-terminal half, but not the C-terminal half, of BspR were localized at the nucleus (Fig 4B), even though the level of expression of the C-terminal half was similar to that of the N-terminal half (Fig 4D). We also obtained similar results from the introduction of these vectors into HeLa cells (S4 Fig). These results clearly demonstrate that BspR has the ability to localize into the nucleus under the direction of its N-terminal 100 aa residues.

## Discussion

In this study, we have demonstrated that BspR can be translocated into the host cells via the T3SS using EPEC as host bacteria (Figs 2 and 3). The results of domain mapping using various truncated derivatives of BspR indicated that the T3SS-mediated extracellular secretion signal is included in the N-terminal 54 aa residues of BspR (Figs 1B and 3B). In contrast, the N-terminal 150 aa residues were sufficient for the maximal translocation of BspR into the host cells (Fig 3C). Further, the exogenous *bspR* expression in COS-7 cells clearly demonstrated that BspR is transported into the nucleus via its N-terminal 100 aa residues (Fig 4). Collectively, these finding reveal that BspR is not only a transcriptional regulator in the bacterial cytosol, but also an effector that is translocated into the host nucleus.

Iron starvation is a key signal for the expression of the *Bordetella* T3SS [9] and deficient in BspR was shown to hyper-produce type III secreted proteins, even in the presence of a high concentration of iron. Thus, iron-responsive unknown factors might properly modulate the endogenous expression of BspR. To eliminate the gene dosage effect of BspR on the *Bordetella* T3SS function, we evaluated the translocation of BspR into host cells by the EPEC T3SS. Indeed, the EPEC T3SS was not affected by the exogenous expression of BspR-CyaA (S3 Fig). Translocation of BspR into the host cells was dependent on a functional T3SS in EPEC, since the T3SS-deficient strain of EPEC was unable to translocate BspR into the host cells (Figs 2 and 3). In our previous study, we showed that the EPEC Map effector was translocated into the host cells via the *Bordetella* T3SS [10]. It has been reported that *Yersinia* effector YopE is delivered into the extracellular milieu via the Xanthomonas T3SS [27], and *Pseudomonas* effectors AvrB and AvrPto are recognized by the *Yersinia* T3SS [28]. Thus, the function of bacterial T3SSs appears to be interchangeable.

In eukaryotic cells, proteins with a molecular mass less than 20–40 kDa are able to passively diffuse into the nucleus. However, cytosolic proteins with a molecular mass greater than 40–60 kDa are translocated into the nucleus in a manner regulated by a nuclear localization signal (NLS) that is generally composed of a short stretch of basic positively charged amino acids (especially lysine and arginine) [29]. NLS-containing proteins can be recognized by importins and the resulting complex is transported into the nucleus through the nuclear pore complex. Interestingly, it has been reported that several bacterial effectors can be translocated into the nucleus by their conserved NLSs [30]. For example, the *Ralstonia solanacearum* effector PopB contains a bipartite NLS (KRKR and KKKKKR) [31], and the *Ralstonia* effector PopP2 also has the capacity for nuclear localization via its N-terminal bipartite NLS (RRRR and RRQRQ) [32]. BspR is a 20-kDa protein (aa 1–191) that is delivered into the host cells via the T3SS [9] and is theoretically able to enter the nucleus passively. Our data clearly showed that the N-terminus of BspR (aa 1–100) is sufficient for nuclear translocation, and that the N-terminal-truncated BspR is unable to localize to the nucleus and is preferentially targeted to the cytoplasmic region. However, we could not detect the NLS in N-terminal BspR using a Web-based prediction program (https://www.predictprotein.org/). Collectively, these findings suggest that BspR is translocated into the nucleus by a non-classical NLS located at the N-terminal region.

A BLAST search showed that BspR is highly conserved among *B*. *bronchiseptica*, *B*. *pertussis*, and *B*. *parapertussis* (~ 97% identity), but not in other *Bordetella* species. Interestingly, *B*. *bronchiseptica* BspR shares 54% amino acid sequence identity with the hypothetical protein NH784_014471 (aa 1–190; YP_008028542) from *Achromobacter xylosoxidans* (S5 Fig). Interestingly, genes encoding BspR and NH784_014471 are located adjacent to the locus of the type III secretion apparatus (S6 Fig). Furthermore, an extracytoplasmic function (ECS) sigma factor BtrS [8] and the hypothetical protein BB1640 exhibit 65% identity with NH784_014481 and 61% identity with NH784_014421, respectively (S6 Fig). Expression of the T3SS genes is regulated by an alternative sigma factor BtrS in *B*. *bronchiseptica* [8]. These findings suggest that BspR homologue NH784_014471 and BtrS homologue NH784_014481 are also involved in the regulation of T3SS in *A*. *xylosoxidans*. *Bordetella* and *Achromobacter* species belong to the family of *Alcaligenaceae*. Therefore, it is suggested that some of the *Bordetella* and *Achromobacter* species acquired the T3SS gene cluster and its regulatory genes by horizontal transfer during the evolutionary process.

As described in detail previously [9], we failed to detect an interaction between BspR and other bacterial factors, including an anti-sigma factor for BtrS. Instead, we observed that the production of BvgA in the *bspR* mutant strain was significantly higher than that in the wild-type *B*. *bronchiseptica* [9]. In *Bordetella* species, a number of virulence genes are coordinately regulated by a two-component signal transduction system, BvgA and BvgS. These findings suggest that BspR governs the regulation of T3SS and other bacterial factors by altering the BvgA content in the bacterial cytosol, rather than via an anti-sigma factor for BtrS.

Our *in vivo* infection study showed that BspR plays key roles in the *Bordetella* virulence [9]. In this study, we showed that BspR is translocated into host cells via the T3SS and is localized into the nucleus via a non-classical NLS located at the N-terminus (aa 1–100), even though BspR functions as a global regulator in the bacterial cytosol [9]. Herein, we propose that BspR is a dual functional effector in both prokaryotic and eukaryotic cells. Further studies are needed to clarify the precise roles played by BspR in the functioning of T3SS and the host-pathogen interaction during *Bordetella* infection. The multifunctional characteristics of BspR in both bacteria and host cells should provide new insight into how *Bordetella* species modulate the expression of virulence genes and the host cell signaling pathways in response to *in vivo* environmental conditions.

## Supporting Information

S1 Fig The exogenous expression of BspR affects CyaA-based translocation assay in *Bordetella*. **(A)** Construction of expression vectors for CyaA fusion proteins. **(B)** HeLa cells were infected with the *Bordetella* S798 Δ*bteA* Δ*cyaA* strain harboring pBteA-48, pBspR-FL, pBspR-150, pBspR-100, or pBspR-54 and then translocation of the CyaA fusion protein via the *Bordetella* T3SS into the infected cells was measured using a cAMP enzyme immunoassay. The values represent the means ± SD from three independent experiments. Note that elevated cAMP level was detected in the HeLa cell lysates infected with the *Bordetella* producing BteA (1–48 a. a.) fused with CyaA, since effector BteA does not affect the T3SS function of *Bordetella* S798 Δ*bteA* Δ*cyaA* strain. Note that an endogenous adenylate cyclase activity was disrupted in the *Bordetella* S798 Δ*bteA* Δ*cyaA* strain. Except for the introduction of pBteA-48, certain levels of cAMP were unable to be detected in double mutant strain.
(EPS)

S2 Fig The exogenous expression of BspR affects the TEM-1-based translocation assay in *Bordetella*. **(A)** HeLa cells were infected with the *Bordetella* S798 Δ*bteA* strain harboring pTEM-1 (TEM-1), pBopC-N3-TEM-1 (BteA-TEM-1), or pRK415-BspR-TEM 1 (BspR-TEM-1) and then stained with CCF2-AM solution. Stained cells were analyzed under a fluorescence microscope. Arrowheads show the fluorescence signal shift to blue from green. Note that BteA-TEM-1, but not BspR-TEM-1, is translocated into the host cells. **(B)** The *B*. *bronchiseptica* S798 Δ*bteA* strain harboring pTEM-1 (TEM-1) or pRK415-BspR-TEM 1 (BspR-TEM-1) was grown in SS medium under vigorous shaking at 37°C for 18 h. The secreted proteins isolated from the bacterial culture supernatants were separated by SDS-PAGE and stained with CBB.
(EPS)

S3 Fig The exogenous expression of BspR does not affect the EPEC T3SS. EPEC strains harboring the full length or truncated versions of BspR fused with CyaA (see Fig 3A) were inoculated in LB and incubated at 37°C for 16 h without shaking. The overnight culture was diluted in DMEM and then the EPEC cultures were grown at 37°C under an atmosphere of 5% CO_2_ for 6 h without shaking. The EPEC-secreted proteins prepared from the bacterial culture supernatants were resolved by SDS-PAGE and stained with CBB. Note that the band intensities of the type III secreted proteins EspA, EspB, and EspD were not affected by the exogenous expression of BspR.
(EPS)

S4 Fig BspR is translocated into the nuclei of host cells. **(A)** The mammalian expression vector for BspR production was introduced into HeLa cells. 24 h after transfection, HeLa cells were fixed and stained with anti-FLAG antibodies to detect BspR (red) and DAPI for nuclei (green). **(B)** The numbers of FLAG-positive nuclei were scored by examining 100 cells per coverslip under a fluorescence microscope. HeLa cells transfected with pC-BspR-FL, pC-BspR-NT, and pC-BspR-CT were randomly picked from the immunofluorescence micrographs **(A)**, and the percentages of cells showing nuclear translocation of BspR were determined. Percentages were based on a count of 100 cells, and the values represent the means ± SD from three independent experiments. *, *p* < 0.05. **(C)** pC-BspR-FL (FL), pC-BspR-NT (NT), and pC-BspR-CT (CT) were introduced into HeLa cells. 24 h after infection, the BspR-FLAG fusion protein was detected by immunoblot analysis of HeLa cell lysate samples.
(TIF)

S5 Fig Amino acid sequence identity between BspR from *B*. *bronchiseptica* and the hypothetical protein NH784_014471 from *Achromobacter xylosoxidans*. (EPS)

S6 Fig Gene clusters of the type III secretion system and its regulatory loci in *B*. *bronchiseptica* and *Achromobacter xylosoxidans*. Genetic maps were illustrated in accordance with data obtained from the SSDB (Sequence Similarity DataBase) at the Kyoto Encyclopedia of Genes and Genomes (http://www.kegg.jp/kegg/ssdb/).
(EPS)

S1 Text Construction of plasmids for the production of BteA-CyaA and BspR-TEM-1 fusion proteins in *Bordetella*. (DOCX)
