# Supplementary figures and images for: The Bordetella Secreted Regulator BspR Is Translocated into the Nucleus of Host Cells via Its N-Terminal Moiety: Evaluation of Bacterial Effector Translocation by the Escherichia coli Type III Secretion System
Source: PLoS One. 2015 Aug 6;10(8):e0135140. doi: 10.1371/journal.pone.0135140 (PMC4527748; doi:10.1371/journal.pone.0135140)

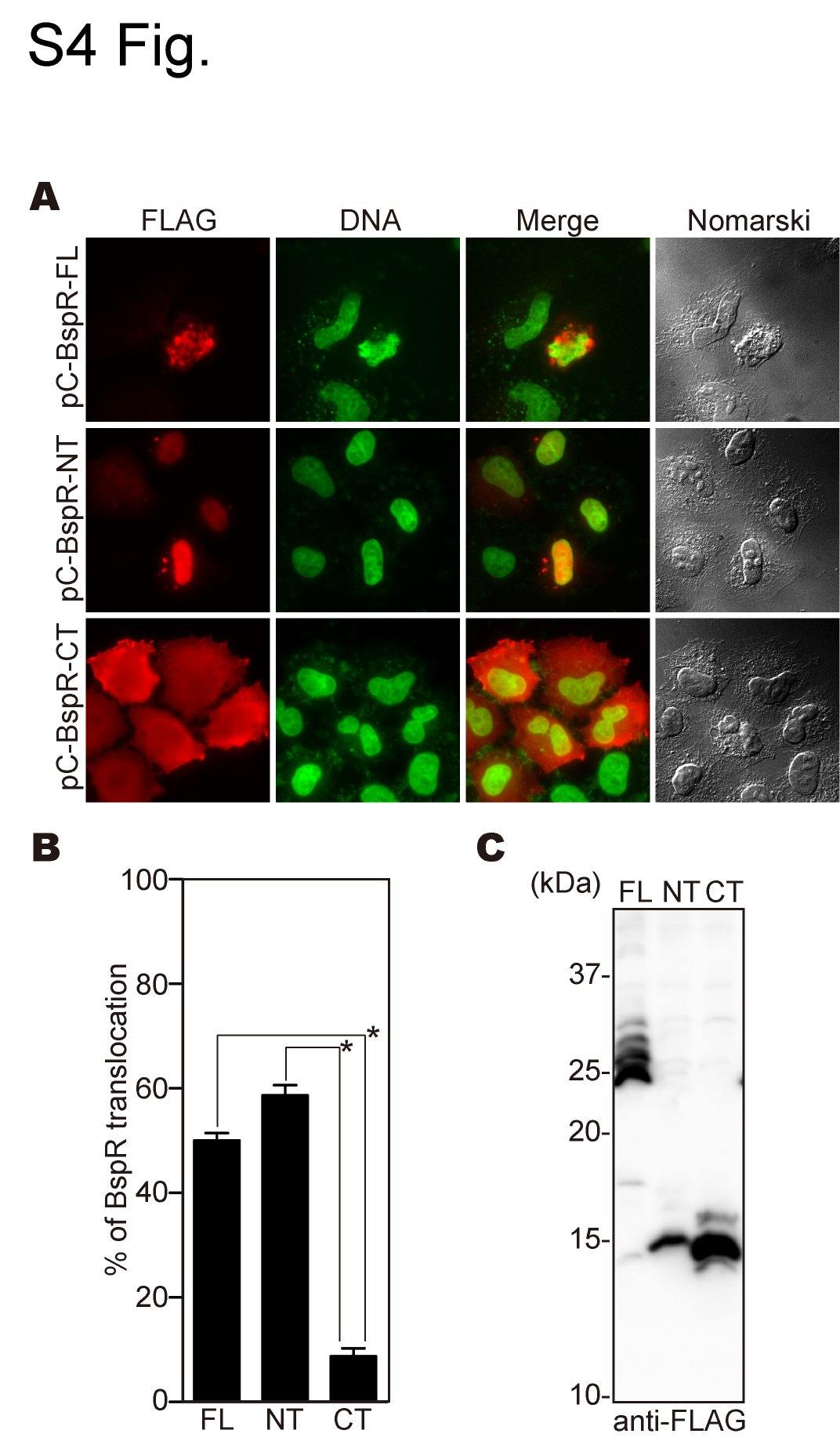

Supplement: S4 Fig — (A) The mammalian expression vector for BspR production was introduced into HeLa cells. 24 h after transfection, HeLa cells were fixed and stained with anti-FLAG antibodies to detect BspR (red) and DAPI for nuclei (green). (B) The numbers of FLAG-positive nuclei were scored by examining 100 cells per coverslip under a fluorescence microscope. HeLa cells transfected with pC-BspR-FL, pC-BspR-NT, and pC-BspR-CT were randomly picked from the immunofluorescence micrographs (A), and the percentages of cells showing nuclear translocation of BspR were determined. Percentages were based on a count of 100 cells, and the values represent the means ± SD from three independent experiments. *, p < 0.05. (C) pC-BspR-FL (FL), pC-BspR-NT (NT), and pC-BspR-CT (CT) were introduced into HeLa cells. 24 h after infection, the BspR-FLAG fusion protein was detected by immunoblot analysis of HeLa cell lysate samples. (TIF) [file pone.0135140.s004.tif]
