# Supplementary material for: The Bordetella Secreted Regulator BspR Is Translocated into the Nucleus of Host Cells via Its N-Terminal Moiety: Evaluation of Bacterial Effector Translocation by the Escherichia coli Type III Secretion System
Source: PLoS One. 2015 Aug 6;10(8):e0135140. doi: 10.1371/journal.pone.0135140 (PMC4527748; doi:10.1371/journal.pone.0135140)
Supplement: S1 Text — (DOCX) [file pone.0135140.s007.docx]

**S1 Text**

**Construction of a plasmid for the production of BteA-CyaA fusion protein in *Bordetella***

In the previous study, we demonstrated that 48 aa residues of N terminal BteA were sufficient for extracellular secretion and translocation into host cells [1]. For this reason, we constructed an expression vector producing BteA N-terminal 48 aa residues fused with CyaA used as a positive control. A 2.0-kbp fragment encoding *bteA* was amplified by PCR with B1-*bteA*-comp (5'-AAAAAGCAGGCT-GGTCACATATGCTGACCT-3') and 3-*bteA*-gs (5'-AGAACCGCCACC-TGCGCGTAGATTCAGCGCCG-3') primers using S798 genomic DNA as a template. A DNA fragment encoding the catalytic domain (N-terminal 400 amino acid residues) of *B.* *pertussis* CyaA was amplified with 5-*cyaA*-gs (5'-GGTGGCGGTTCT-CAGCAATCGCAT-CAGGCTGG-3') and B2-*cyaA* (5'-AGAAAGCTGGGTCTA-GTCATAGCCGGAATCCTGGC-3') primers using pMS109 [2] as a template. Both *bspR* and *cyaA* fragments were ligated by adapter PCR method with B1-*bteA*-comp and B2-*cyaA*. The resulting *bteA*-*cyaA* was cloned into pDONR221 to obtain pDONR-BteA-FL by BP Clonase reaction in the Gateway cloning system (Invitrogen). To obtain plasmids encoding truncated versions of BteA fused with CyaA, inverse PCR was performed with the primers KpnI-gs-cyaA (5'-gg-ggtacc-GGTGGCGGTTCT-CAGCAATC- 3') and p74-100-bla-KpnI (5'-gg-ggtacc-GCTGGCCACGCTGCGCACG-3') using circular pDONR-BteA-FL as a template to obtain pDONR-BteA-48 that produces the N-terminal 48 aa residues of BteA fused with CyaA. To allow expression of the *bteA-cyaA* fusion gene under the control of the *fha* promoter and the *rrnB* terminator, pDONR-*fha*P [1], pDONR-BteA-48, pDONR-*rrnB* [1], and *Bordetella* vector pRK415 R4-R3-F [1] were mixed and treated with LR Clonase Plus (Invitrogen) to clone the *fha* promoter, *bteA-cyaA* gene, and *rrnB* terminator into pRK415 R4-R3-F using the MultiSite Gateway system (Invitrogen), and the resulting plasmid was designated pBteA-48.

**Construction of a plasmid for the production of BspR-TEM 1 fusion protein in *Bordetella***

For the expression of BspR fused with TEM 1 β-lactamase, *bspR* was cloned into the plasmid pCX340 [3] as follows. A 0.9 kbp fragment encoding *bspR* was amplified by PCR with the primers 5-bb1639-NdeI (5'-GGAATTCCATATGACTCTCCGCGTTGACGGCGC-3') and 3-bb1639-KpnI (5'-GGGGTACCCAGGTGGTGCGCAAGGACCT-3') using S798 genomic DNA as a template. The resulting fragment was digested with NdeI and KpnI and then cloned into similarly digested pCX340. The resulting plasmid was designated pCX340-BB1639 that produces the full length BspR fused with TEM 1. The *bspR* fused with TEM 1 β-lactamase gene (*bla*) was amplified by PCR with the primers B1-bb1639-comp (5'-AAAAAGCAGGCTACTCTCCGCGTTGACGGCGC- 3') and B2-*bla*-pCX340 (5'-AGAAAGCTGGGTTTACCAATGCTTAATCAGTG-3') using circular of pCX340-BB1639 as a template and then *att*B1 and *att*B2 recognition sites were added to the 5' and 3' flanking sites of the *bspR*-*bla* fused gene, respectively, using the adaptor PCR method in the Gateway cloning system (Invitrogen). The resulting *bspR*-*bla* was cloned into pDONR221 to obtain pDONR221-bb1639-FL-*bla* by BP Clonase reaction in the Gateway cloning system (Invitrogen). To allow expression of the *bspR-bla* fusion gene under the control of the *fha* promoter and the *rrnB* terminator, pDONR-*fha*P [1], pDONR221-bb1639-FL-*bla*, pDONR-*rrnB* [1], and *Bordetella* vector pRK415 R4-R3-F [1] were mixed and treated with LR Clonase Plus (Invitrogen) to clone the *fha* promoter, *bspR-bla* gene, and *rrnB* terminator into pRK415 R4-R3-F using the MultiSite Gateway system (Invitrogen), and the resulting plasmid was designated pRK415-BspR-TEM 1. pTEM 1 [1] and pBopC-N3-TEM 1 [1] were used as negative and positive control vectors, respectively.

Reference

1. Kuwae A, Matsuzawa T, Ishikawa N, Abe H, Nonaka T, Fukuda H, et al. BopC is a novel type III effector secreted by *Bordetella* *bronchiseptica* and has a critical role in type III-dependent necrotic cell death. J Biol Chem. 2006;281: 6589–6600. doi:10.1074/jbc.M512711200

2. Miki T, Shibagaki Y, Danbara H, Okada N. Functional characterization of SsaE, a novel chaperone protein of the type III secretion system encoded by *Salmonella* pathogenicity island 2. J Bacteriol. 2009;191: 6843–6854. doi:10.1128/JB.00863-09

3. Charpentier X, Oswald E. Identification of the secretion and translocation domain of the enteropathogenic and enterohemorrhagic *Escherichia* *coli* effector Cif, using TEM-1 β-Lactamase as a new fluorescence-based reporter. J Bacteriol. 2004;186: 5486–5495. doi:10.1128/JB.186.16.5486-5495.2004
